# Combined use of high doses of vasopressin and corticosteroids in a patient with Crohn’s disease with refractory septic shock after intestinal perforation: a case report

**DOI:** 10.1186/s13256-017-1456-3

**Published:** 2017-11-13

**Authors:** Salvatore Notaro, Marcello Sorrentino, Aniello Ruocco, Annalisa Notaro, Antonio Corcione, Patrizia Murino, Eugenio Piscitelli, Marianna Tamborino

**Affiliations:** 1 Intensive Care Unit, AORN dei colli Vincenzo Monaldi Hospital, Naples, Italy; 2 Intensive Care Unit, Fatebenefratelli Hospital, Naples, Italy; 3 Department of Pharmacology, Federico II University of Naples, Naples, Italy

**Keywords:** Vasopressin, Corticosteroid, Refractory septic shock

## Abstract

**Background:**

In this article, we present a clinical case of refractory septic shock resulting from intestinal perforation treated with high doses of vasopressin and hydrocortisone during emergency surgery.

The use of such high doses of vasopressin for this type of shock is not described in the literature.

**Case presentation:**

A 49-year-old white woman with grade III obesity, Crohn’s disease, and an intestinal perforation presented with refractory septic shock. Initially, a low dose of vasopressin was used. Then, the dosage was increased to 0.4 U/minute; in the literature, this is defined as “salvage therapy.” This therapy consists of an initial load followed by a continuous infusion of hydrocortisone.

**Conclusions:**

The significant increase in her cardiac index and stroke volume index resulted in an improvement in peripheral resistance, gas exchange, and urine output and a decrease in her heart rate, interleukin-6 level, and tumor necrosis factor-α level. The administration of high doses of vasopressin and corticosteroids was demonstrated to be safe for the immune system, to reduce the systemic inflammatory response, and to have direct cardiovascular effects. Further studies are required to examine the use of vasopressin as an initial vasopressor as well as its use in high dosages and in combination with corticosteroids.

## Background

Crohn’s disease is a chronic inflammatory disease that affects the intestinal wall in any section of the alimentary canal.

Macrophages release cytokines; the most commonly produced cytokine is interleukin (IL)-1, which causes inflammation with ulceration of the mucous membrane. This ulceration can degenerate into a perforation and finally become peritonitis, which rapidly evolves until septic shock develops. Septic shock is characterized by abnormal pro-inflammatory and anti-inflammatory responses, microvascular damage, hypoxia, and vasodilation with hypotension.

Vasopressin (VP) should be added to norepinephrine (NE) treatment to either increase mean arterial pressure (MAP) or to reduce the NE dosage. VP doses > 0.04 units/minute should be reserved for salvage therapy or, upon failure, to achieve adequate MAP together with other vasopressors.

Glucocorticoids can reduce the risk of the evolution of sepsis into septic shock or improve the state of shock, which reduces mortality [1].

We describe a case of intestinal perforation in a patient with refractory septic shock as a result of treatment with NE who was treated with VP dosages higher than those described in the literature as “salvage therapy”, together with a hydrocortisone load followed by continuous infusion. This treatment allowed us to gradually reduce the NE level and keep the patient alive until surgical infection control was achieved, without side effects such as mesenteric ischemia, heart failure, skin and digital necrosis, or decreased cardiac output.

No clinical cases in the literature have described the use of high-dose VP in combination with continuous infusion of intravenously administered corticosteroids or the mutual interaction of the two drugs in a patient with refractory septic shock.

## Case presentation

A 49-year-old white woman affected by Crohn’s disease who recently underwent a left hemicolectomy with enterostomy, was treated for septic shock resulting from intestinal perforation. Her height was 150 cm, weight 130 kg, and she had a body mass index (BMI) of 57 which is class III obesity.

Her clinical characteristics included sleepiness, 90% oxygen saturation, a fraction of inspired oxygen (FiO_2_) of 100%, cyanotic and cold limbs, hypothermia, and a large abdomen that compromised lung ventilation and perfusion of the splanchnic organs. Her most severe symptoms included the following:

- Her blood pressure was 50/30 mmHg and her heart rate was 140 beats per minute (bpm) with many ventricular triplets. She was also anuric.
- Blood gas analysis showed acidosis with the following values: a pH of 7.10, partial pressure of carbon dioxide (pCO_2_) 60 mmHg, partial pressure of oxygen in arterial blood (PaO_2_) 50 mmHg, base excess (BE) −10, bicarbonate (HCO_3_) 15 mmol/L, lactate 15 mmol/L, potassium ion (K+) 2.3 mEq/L, sodium ion (NA+) 120 mEq/L, central venous saturation of oxygen (ScvO_2_) 45%, and venous-to-arterial difference of carbon dioxide (delta pCO_2_) 15.
- Laboratory tests showed the following: leucocytosis, anemia, thrombocytopenia, procalcitonin 70 ng/mL, alanine aminotransferase (ALT) 250 U/L, aspartate aminotransferase (AST) 180 U/L, lactate dehydrogenase (LDH) 350 U/L, blood urea nitrogen (BUN) 150 mg/dL, creatinine 2.3 mg/dL, IL-6 300 pg/mL, and tumor necrosis factor-alpha (TNF-α) 200 pg/mL.
- Echocardiography showed widespread hypokinesia, an ejection fraction of 25%, and diastolic dysfunction with a tricuspid annular plane systolic excursion (TAPSE) of 10 mm.
- Her lungs were wet and had an interstitial pattern with moderate bilateral pleural effusion.
- A computed tomography (CT) scan of her abdomen showed pneumoperitoneum with free fluid in her abdomen and particulate matter that extended between the intestinal loops for approximately 30 to 40 cm.

Due to the severity of the situation, she was prepared for an emergency laparotomy and underwent a treatment known as “early goal-directed therapy”.

After fluid resuscitation with 30 mL/kg of crystalloid and antibiotic administration for abdominal sepsis with Merrem (meropenem) 2 g + tigecycline 100 mg + caspofungin 70 mg, we performed external heating and fluid infusions without obtaining the target MAP. Owing to the failure to respond to the fluid load challenge, we started NE infusion at 0.05 mcg/kg per minute, increasing the dosage until it reached 1 mcg/kg per minute; despite the increased dosage, good peripheral perfusion was not achieved.

Then, we induced rapid sequence anesthesia (succinylcholine 100 mg + fentanyl 100 mcg + midazolam 7 mg) to allow intubation, and anesthesia was maintained with 0.1 mcg/kg per minute of remifentanil and 0.2 mg/kg of sevoflurane and cisatracurium. Protective mechanical ventilation was set to 6 mL/kg tidal volume (VT), with a positive end-expiratory pressure (PEEP) of 8. Intraoperative hemodynamic monitoring via an EV1000 (Edwards Lifesciences, Irvine, California, USA) indicated cardiac index (CI), stroke volume variation (SVV), stroke volume index (SVI), and systemic vascular resistance index (SVRI) values of 1.2, 17, 20, and 400, respectively. NE infusion was increased to a maximum value of 2.5 mcg/kg per minute without obtaining a MAP > 65 mmHg. Hence, we added VP, starting with 0.02 units/minute and increasing to a dose of 0.4 units/minute. In addition, we administered hydrocortisone in an initial load of 1 g and then via an infusion of 0.2 mg/kg per hour.

During the surgery, a perforation was found 80 cm from the ileocecal valve ileum, with an abscess and multiple adhesion areas of fecal material between the bowel loops. A bowel resection was performed with a latero-lateral anastomosis and viscerolysis. After 2 hours, our patient had a MAP ≥ 65 mmHg, an ScvO_2_ ≥ 75 mmHg, a reduction of arterial lactate, restoration of adequate hemodynamic properties, and improved cardiac output, stroke volume, and SVV, as well as a gradual reduction of noradrenaline after 4 hours.

At the end of surgery, she received a stable VP dose of 0.04 U/minute; continued hemodynamic monitoring showed improvements in the CI to 2.3, SVV to 12, SVI to 30, and SVRI to 750. In our Intensive care unit (ICU), she exhibited a significant reduction in inflammatory cytokine levels, including a reduction in IL-6 to 50 pg/mL and a reduction in TNF-α to 75 pg/ mL. She was weaned from vasoactive therapy at 12 hours; we performed continuous hydrocortisone infusion for 24 hours with a gradual reduction of the dose over the following 5 days until suspension of the therapy.

One week after surgery, she was weaned from ventilation and moved to our Surgery department. At 28 days, she was discharged. She underwent monthly follow-up evaluations, which showed remission of the inflammatory disease and the absence of infectious or cardiovascular complications.

## Discussion

VP is an endogenous stress hormone that is produced in the hypothalamus and released from the posterior pituitary gland. Its receptors include V1, V2, and V3. VP binds G-protein-coupled V1 receptors on vascular smooth muscle to induce vasoconstriction, while nitric oxide induces vasodilation of the coronary and pulmonary vessels. The V2 receptor enables water reabsorption by the distal nephron [2], while interaction with V3 induces the release of adrenocorticotropic hormone (ACTH). Moreover, VP binds to oxytocin and purinergic receptors and mediates vasodilation via stimulation of the nitric oxide pathway in pulmonary, coronary, and cerebral endothelial cells.

At low doses, VP has vasodilatory effects, while at high doses, it has vasoconstrictor effects. Early initiation of VP therapy could decrease the incidence of new onset arrhythmias [3]. VP may act on the baroreceptor reflex to control the accelerator center of the cardiac brainstem. Ischemic effects in the mesentery and skin may be prevented by an adequate volume status; intensive hemodynamic monitoring can produce a rapid rebalance of the volume status [4].

A recent randomized, controlled, concealed trial of VP versus NE suggested that low-dose VP may decrease mortality in patients with less severe sepsis [5]. Hydrocortisone therapy probably reduces both the duration and the dosage of VP infusion [6]. In septic shock pro-inflammatory cytokines may have a downregulatory effect on V1A receptor expression by reducing sensitivity to endogenous vasopressin or administered in continuous infusion [14]. Glucocorticoids may reverse the cytokine-mediated down-regulation of V1 receptors located in the vascular endothelium [6–15]. When combined with VP, the receptors could produce synergistic effect to maintain the MAP.

Low-dose VP infusion in addition to corticosteroid administration significantly decreases mortality and organ dysfunction compared to that associated with the combination of corticosteroids and NE [7]. In the abovementioned case, we used high-dose VP in combination with hydrocortisone. This process was lifesaving in a critically ill patient who had an inadequate response to NE. Subsequently, our patient underwent emergency surgery, and septic shock was ameliorated.

High doses of glucocorticoids have genomic and non-genomic effects and exhibit a peak that can appear between the first minute after intravenous administration and the following 3 hours. These effects are exploited to treat many rheumatic diseases and spinal cord injuries [8]. Recent studies have shown that hydrocortisone decreases specific circulating B and T cell lymphocytes subsets. While transcriptome profiling has revealed down-regulation of NF-kB signaling, apoptosis and cell death signaling transcripts precede lymphocyte population changes, with activation of natural killer (NK) cells and glucocorticoid receptor signaling transcripts [9].

VP possesses a potent anti-inflammatory capacity; phosphoinositide 3-kinase and its downstream Akt activator contribute to the endogenous anti-inflammation capacity, while macrophages show a reduced release of cytokines (TNF-α and IL-6) and chemokines [10].

## Conclusions

In conclusion, although we safely used a VP rescue dosage (0.4 U/minute) with increased corticosteroid levels, based on recent clinical trials, VP may exhibit the greatest benefit for septic shock treatment when used early in patients with less severe shock.

Titration of VP in rescue doses with the addition of high doses of hydrocortisone enabled suspension of NE and reduction of the side effects and the duration of VP infusion.

We believe that the combined use of the two drugs had synergistic effects on the endocrine, vascular, and immunological systems, strengthened the anti-inflammatory effects, and drastically reduced the cytokine storm, thus preventing further deterioration and improving hemodynamic properties, which allowed us to eradicate the focus of the infection and gain time for surgical treatment.

In clinical practice, many patients are treated with these two medications, but few studies on their interaction have been published.

The early use of VP may have benefits [11] similar to Rivers’ early goal-directed therapy [12]. The use of VP as the initial vasopressor therapy, with or without corticosteroids, requires further study [13]. Use of higher doses of VP and the dosing limits should be further examined to assess the efficacy and safety of this approach.

## Abbreviations

CI: Cardiac index
IL: Interleukin
MAP: Mean arterial pressure
NE: Norepinephrine
ScvO_2_: Central venous saturation of oxygen
SVI: Stroke volume index
SVRI: Systemic vascular resistance index
SVV: Stroke volume variation
TNF-α: Tumor necrosis factor-alpha
VP: Vasopressin
